# Do Women in Nepal Like Playing a Mobile Game? MANTRA: A Mobile Gamified App for Improving Healthcare Seeking Behavior in Rural Nepal

**DOI:** 10.3389/fpubh.2021.645837

**Published:** 2021-11-05

**Authors:** Rachya Kayastha, Sonja Mueller, Punam Yadav, Ilan Kelman, Andrei Boscor, Naomi Saville, Abriti Arjyal, Sushil Baral, Maureen Fordham, Gareth Hearn, Patty Kostkova

**Affiliations:** ^1^UCL Centre for Digital Public Health in Emergencies (dPHE), Institute for Risk and Disaster Reduction, University College London, London, United Kingdom; ^2^Institute for Global Health, University College London, London, United Kingdom; ^3^Centre for Gender and Disaster, Institute for Risk and Disaster Reduction, University College London, London, United Kingdom; ^4^Health Research and Social Development Forum (HERD International), Kathmandu, Nepal; ^5^Hearn GeoServe, Ltd., Worthing, United Kingdom

**Keywords:** maternal health, neonatal health, mHealth, LMIC, educational game, knowledge gain, serious games

## Abstract

In Low and Middle Income Countries (LMIC), one of the causes of maternal and child mortality is a lack of medical knowledge and consequently the inability to seek timely healthcare. Mobile health (mHealth) technology is gradually becoming a universal intervention platform across the globe due to ubiquity of mobile phones and network coverage. MANTRA is a novel mHealth intervention developed to tackle maternal and child health issues through a serious mobile game app in rural Nepal, which demonstrated a statistically significant knowledge improvement in rural women. This paper explores the perceptions and usability of the MANTRA app amongst rural women and Female Community Health Volunteers (FCHVs) in Nepal. Despite the challenges of a target user group with limited educational levels and low smartphone experience, all participants viewed the MANTRA app with approval and enthusiasm. They were willing to engage further with the mHealth intervention and to share their experience and knowledge with fellow community members. Participants also showed an increase in awareness of danger signs enabling them to make better informed health decisions in the future. FCHVs viewed the app as a validation tool providing and support for greater impact of their efforts in rural Nepal. Growing mobile ownership, network coverage and availability of smartphones along with acceptance of the prototype MANTRA app in rural communities suggest encouraging prospects for mHealth interventions to be incorporated in the national health infrastructure in Nepal and other LMICs.

## Introduction

“Ensuring healthy lives and promoting well-being for all” is the United Nation's third sustainable development goal (1). It helps to focus efforts to improve maternal health outcomes through novel technological interventions that combat the causes of maternal, neonatal and child deaths, which are concentrated in low and middle-income countries (LMICs) of Asia and Africa (2). Thaddeus and Maine's [(3). p.1096–7] “three delay model” identifies “delay in deciding to seek [medical] care” due to lack of awareness about risk conditions and pregnancy complications as significant in creating complications.

Neonatal deaths are widely caused by premature births, infections, and birth asphyxiation (4) while post-partum hemorrhage, hypertension and sepsis are the major causes of maternal mortality (5). Though such conditions are preventable, lack of access and knowledge of antenatal, delivery and post-partum care may hinder timely delivery of life-saving interventions (6).

Health education around the world is being transformed by the introduction of new technologies for aiding communication and knowledge sharing. Mobile health (mHealth), defined as “mobile computing, medical sensor, and communications technologies for healthcare” (7) is a novel tool which is effective in improving health outcomes. mHealth may empower people in LMICs by helping communities, and especially women, to access knowledge about health seeking, which in turn may lead to improved health behaviors. Thus, mHealth may have potential to alleviate maternal and neonatal mortality (8, 9).

Nepal is a LMIC where maternal and neonatal mortality is still prevalent, with a neonatal mortality rate of 21 per 1,000 live births and a maternal mortality ratio of 239 deaths per 100,000 live births (10, 11). Female Community Health Volunteers (FCHVs) are the primary healthcare education frontline workers established by the Nepalese government in 1988 (12). FCHVs offer maternal health education, encourage antenatal, delivery and postpartum care seeking, provide birth preparedness counseling, identify danger signs in newborns and postpartum women during postpartum visits, and refer on to appropriate health facilities as needed (13). Due to the shortage of skilled health workers (14), FCHVs play an important support role as health educators and mHealth could be an opportunity for further health mobilization in Nepal.

The University College London (UCL) Centre for Digital Public Health in Emergencies (dPHE) developed an intuitive educational mobile app as a learning tool for rural women in Nepal. Maternal and Newborn Technology for Resilience in Rural Areas (MANTRA) was established to bring awareness of risk factors through a serious gaming application (app) pertaining to maternal, child health and geo-hazards. Serious games are digital games designed for education, outreach and training purposes and not just for entertainment (15). The key element of MANTRA was to educate women regarding different risks during pregnancy by enabling them to understand which problems present high risk and what level of care to seek for different problems. It offered FCHVs preliminary hands-on experience with a new technological intervention to improve their maternal and neonatal health knowledge and support overall resilience. Mueller et al. (16) describes the MANTRA mobile app and its development in detail.

The research question and key objectives of this study are to understand the attitudes of women about mobile games, understand the acceptability and usability of the prototype MANTRA intervention by women and FCHVs and to explore their perceptions of knowledge change brought about by the mHealth gaming application. In this paper, we present the study background and context of Nepal (section Background), methodology and data sources (section Methodology), results (section Findings), discussion of the results (section Discussion), and conclusions (section Conclusions).

## Background

### Conceptualization of mHealth

mHealth stems from the evolution of information and communications technology (ICT) creating a unique feature of personalization through mobile devices. It is one of the four pillars of ICT in the healthcare domain (17) and a major focus in the current boom of digital health (18). mHealth is defined as “medical and public health practice supported by mobile devices, such as mobile phones, patient monitoring devices, personal digital assistants, and other wireless devices” (19), mHealth is patient-centric and benefits governments by decreasing healthcare expenditures by delivery of efficient and cost-effective services (20). There are a number of eHealth serious games in the domain of health improving user knowledge and health outcomes (21).

mHealth promotes innovation, stimulating a global strategy for improving maternal and child health while directing nations to integrate such technologies into their national health infrastructure (22). mHealth's role is vital in LMICs for effective utilization of affordable health promotion, education, emergency medical response, treatment compliance and disease management (23, 24).

mHealth in LMICs is mainly applied for training health workers, remote data collection, raising awareness, education and remote monitoring etc. (25). However, organizations render various service elements under the broad umbrella of mHealth. WHO mHealth and ICT framework for reproductive, maternal, newborn and child health (RMNCH) classify their structure under 12 common applications such as short message service (SMS), behavior change communication, Interactive Voice Response, and provider-to-provider communication (26), aiming to provide information and care across the full continuum of maternal health (adolescent through postpartum) and child health (postnatal newborn through childhood).

### Mobile Technology in Nepal

**Figure 1:**
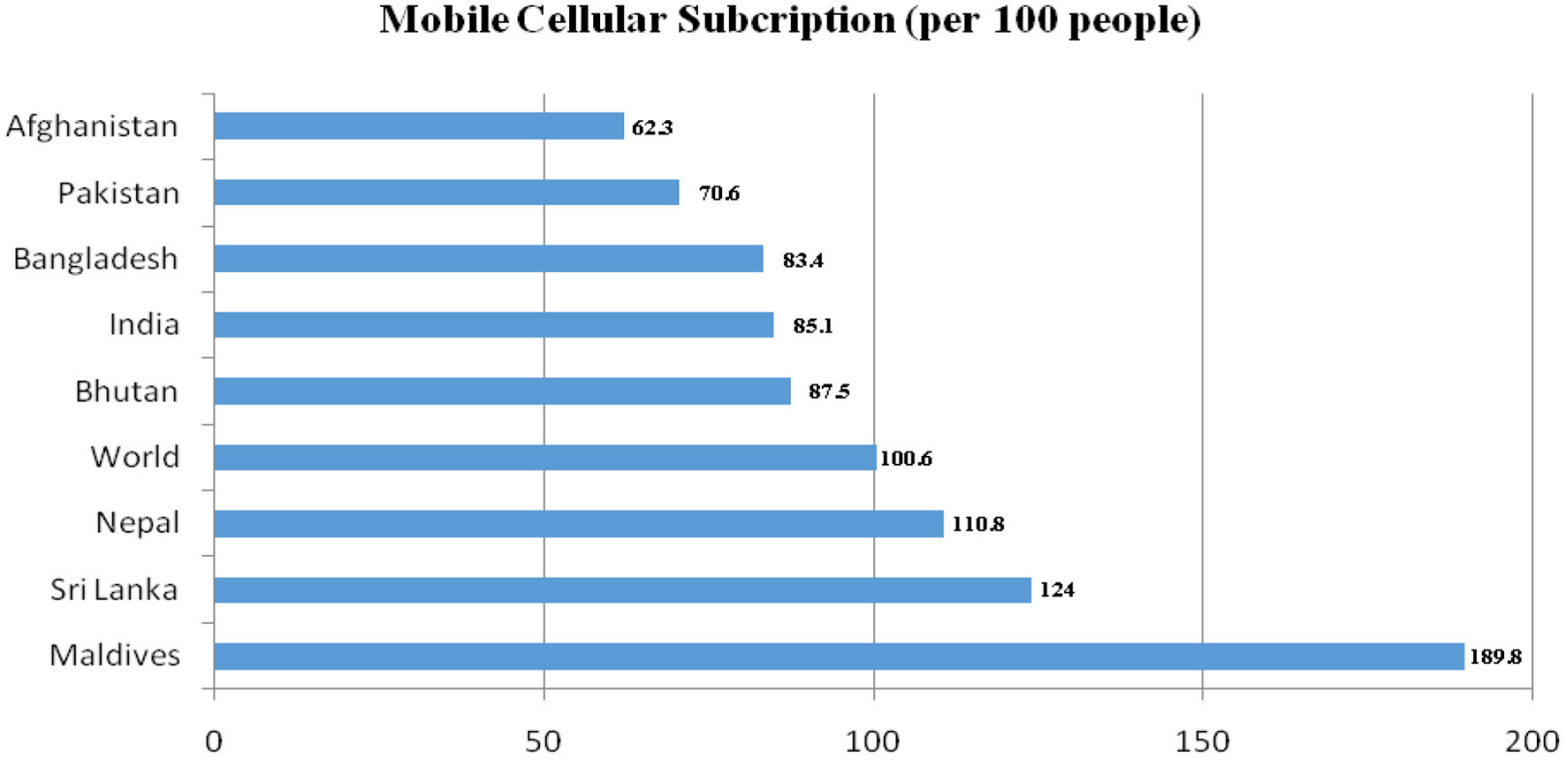
Mobile Cellular Subscriptions in South Asia in 2016. Data used under CC BY-4.0 www.creativecommons.org/licenses/by/4.0 (29).

Nepal Telecommunications Corporation (NTC), a government-owned subsidiary established in 1975 (27), launched the first Global System for Mobile communication (GSM) service in 1999 (28). The monopoly of NTC ended in 2004, opening telecommunications to the private sector. Currently NTC and Ncell Private Limited are the key providers of voice telephony services in Nepal (27). World Bank's 2016 data (Figure 1) ranks Nepal as having the third highest mobile subscriptions in South Asia with ~110 subscriptions per 100 people (29).

**Figure 2:**
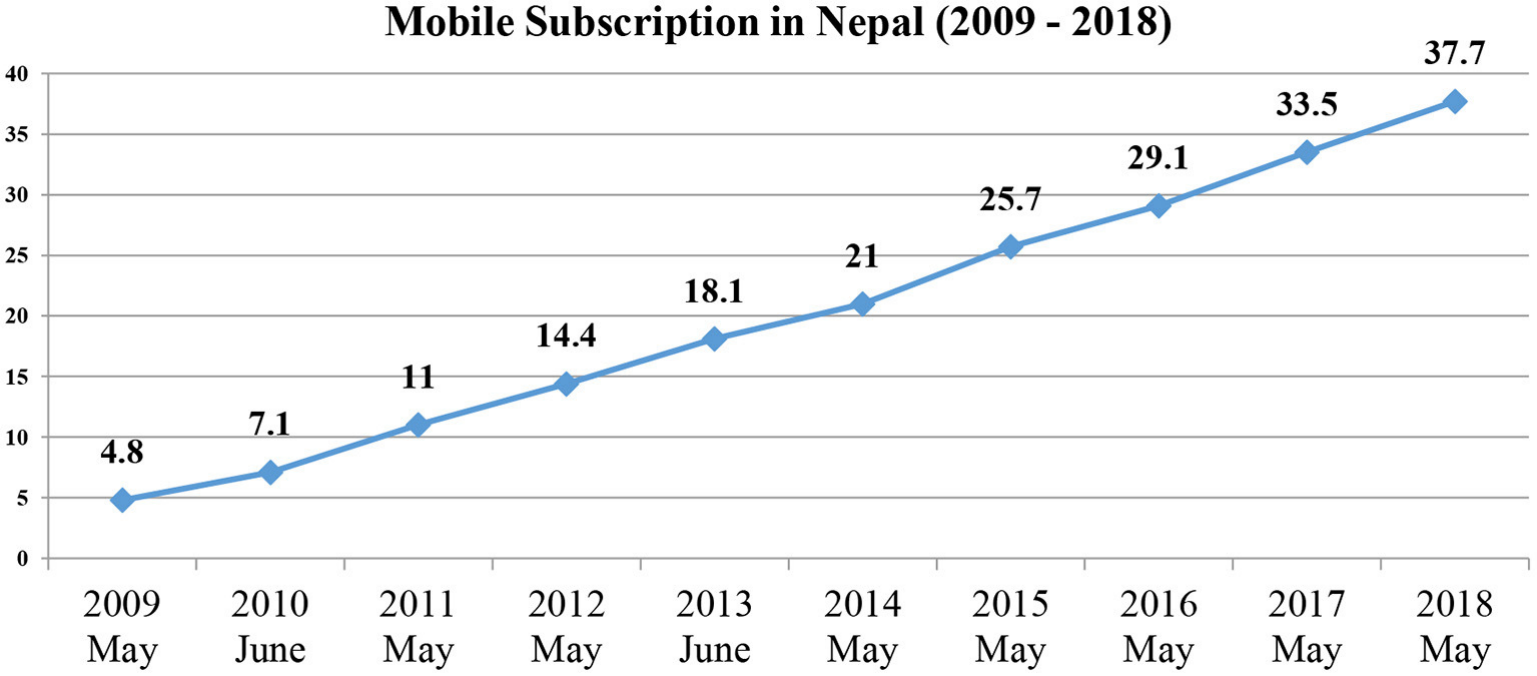
Mobile subscriptions data extrapolated from NTA Reports 2009–2018 [modified from NTA (30)].

Nepal Telecom Authority (NTA), the regulatory governing body for the telecommunications sector in 2018 reported more than 37 million subscriptions (defined as the number of active SIM cards) of mobile telephony services, increasing yearly since 2009 (Figure 2) (30). Based on Nepal's current population estimate of 29 million (31). Figure 2 suggests there may be individuals who own multiple SIMs (32).

**Figure 3:**
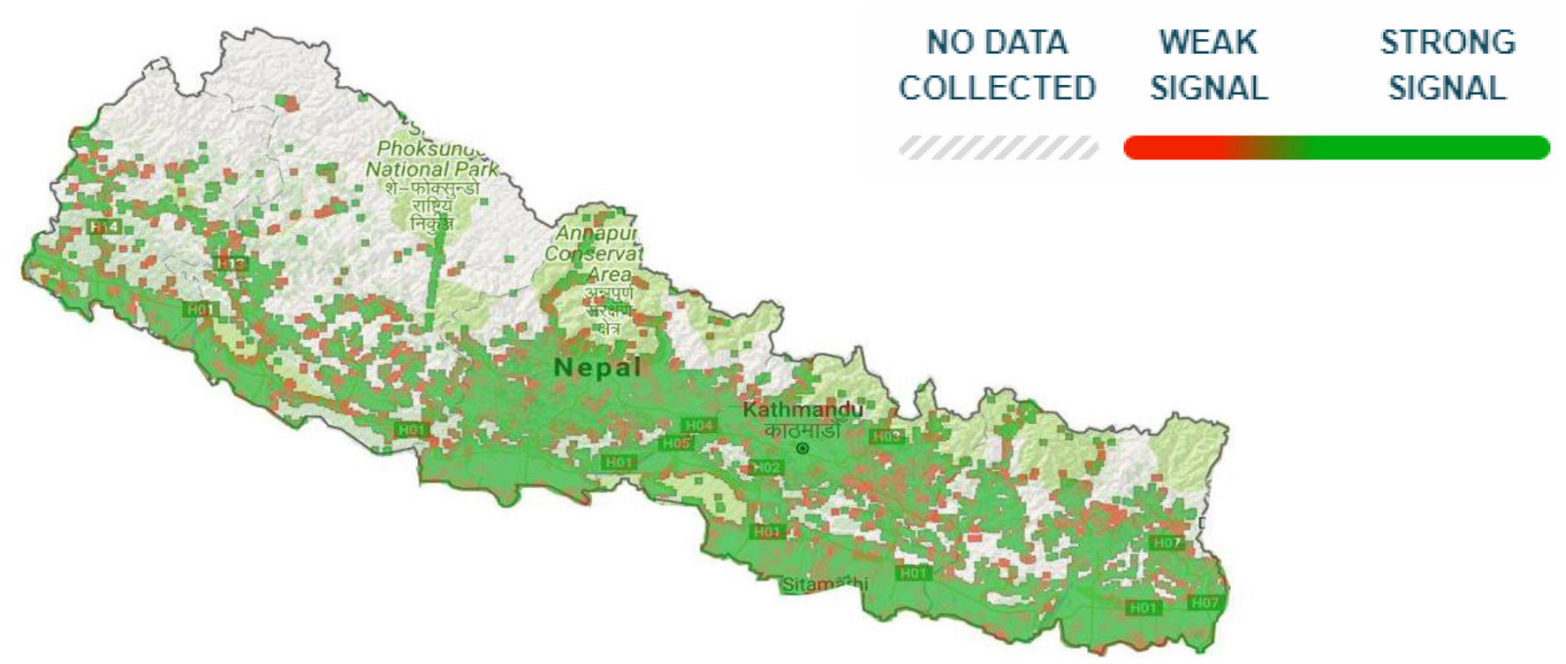
Opensignal 2G/3G/4G coverage map of Nepal (33).

The map of Nepal's network coverage (Figure 3) shows network signal scattered across the country with the strongest signal across urban cities and a majority in the more accessible southern plains belt. However, the northern mountain region lacks full coverage (33). Despite this incomplete coverage, smartphone availability is growing with affordable Chinese brands being introduced in the market (34).

### Serious Games for Health

Mobile learning is an emerging tool attracting interest in all fields of learning and education (35). Games have transformed from a purely entertainment view-point to a concept of serious games that bring change in a player's knowledge, attitude and health by creating attraction from its recreational element (36). An increase in ubiquity and personal ownership of mobiles has led to the introduction of numerous serious gaming apps through various mobile platforms (37). For instance, to educate learners (38–41), train healthcare professionals (42), inform citizens about health (43–45), rehabilitation (46) and management of illness (47, 48).

Urturi et al. (38) mobile app aimed to improve the quality of life of individuals with Autism Spectrum Disorder (ASD). The instructional videos and writing taught participants new ways of dealing with first aid situations. The pilot study tested 10 participants with ASD who reported enjoyment and ease in using the app. Some described having no affinity for games or smartphones. Another observational analysis of 15 participants who played an app aimed at raising awareness about Alzheimer's disease and its effects on families showed behavioral change in participants. The pre- and post- survey evaluations suggest an increase in awareness and new learning of Alzheimer's symptoms (44). However, the sample size of both studies was very small indicating the need for further research to evaluate serious games.

Orjuela et al. (42) study designed a gaming app allowing medical students to practice Cardio-Pulmonary Resuscitation on phones rather than on conventional mannequins. Some students perceived the app as difficult to use, while some preferred the traditional method of teaching using dummies. However, most students found the app related well with their lectures and practical classes, perceiving it as a complementary tool for learning. The eBug project (49, 50) illustrated the opportunities that serious games represent for child learning but also highlighted the challenges of developing such educational initiatives in a low literacy group. This project was expanded by the edugames4all initiative and explored usability challenges for game designers developing games aimed at children (51).

Another example of an interactive game in mHealth is UnderControl, aimed at promoting birth control practices and STI prevention mechanisms for reproductive health. Eleven game testers performed surveys on player experience in difficulty, playability and visual aesthetics. Though participants reviewed the game as engaging, it required adjustments to set challenges based on players' skill levels. The author states, “educational games on reproductive health are very limited on mobile platforms” (43) highlighting the need and potential for such gaming apps.

### mHealth Maternal Interventions in LMICs

There are many mHealth interventions on RMNCH in LMICs (20). Various countries use mHealth as a mode of case management starting from client registration, home visit scheduling, counseling and for follow-up reminders (52–54), with the most common medium being through text or audio. For example, regarding antenatal care (ANC) visits or for sending educational messages (55–58). However, there has been limited research on the educational aspects of mHealth in South Asia (59, 60).

In Jharkhand, India “Mobile for Mother” (MFM) app was launched to aid community health workers (CHWs) and to improve information, data collection systems and increase women's knowledge and health-seeking behaviors. CHWs found MFM to be a systematic source for information, leading to better performance of maternal health-related tasks and building their confidence. However, they perceived mobile phones to bring knowledge change in women but not amongst CHWs (54).

In Ethiopia, researchers provided three mobile phones to each facility with preloaded apps which sent reminders to health-workers regarding scheduled ANC visits, delivery and postnatal care and educational messages on danger signs. Results show the mobile reminders motivated the health-workers to call pregnant women for their scheduled visit assisting in better case management. Their knowledge of danger signs and common illnesses may have also increased because of the weekly educational messages. The app was associated with an increase of ~20% more hospital deliveries and postnatal care in the intervention group compared to the control group (57).

MOTECH program in Ghana focused on improving pregnant and postpartum women's knowledge and awareness of health information. The app also sent automated educational voice messages to pregnant women's mobile phones. This intervention was successful in improving knowledge, awareness and utilization of RMNCH services. The rates of active listening were higher on information of danger signs during pregnancy and post-partum care than on infant care, nutrition and postpartum family planning (60). This suggests women were keen to learn about danger signs or lacked such knowledge, resulting in their interest to learn. If women are able to recognize danger signs, they are more likely to take appropriate healthcare seeking actions (61).

### mHealth in Nepal

The challenging mountainous terrain, limited mobile coverage and widespread poverty of rural populations make mHealth projects challenging in Nepal, but in recent years there have been a number of successes. In collaboration with UNFPA and USAID, Nepal's Ministry of Health (MOH) launched a serious gaming app “Khulduli” in Nepalese language, promoting adolescent sexual and reproductive health awareness. The program is yet to be evaluated, yet the app is the first step toward integrating mHealth into Nepal's health infrastructure. This was a key message in UNFPA's global strategy for women's, children's and adolescent's health (62).

Use of mobile technology for health interventions is relatively new in Nepal. The oldest published study of 2008 describes the use of mobile phones as a primary communication method during medical emergencies (63). Nepal's Demographic and Health Survey carried out in 2011 used tablet PCs for the first time, making substantial improvement in data quality and reduction in data collection time (64). An observational study regarding the role of ICT in medical education elucidates how students enrolled in teaching hospitals use mobile phones for learning by enhancing knowledge through interfaces like Google (65). Kathmandu Medical College Teaching Hospital used mobiles as a hospital management information system. Free-to-use apps like Viber and Dropbox improved their efficiency through instant communication, decision making and cross-country knowledge exchanges (66).

Style et al. (67) on the implementation of an Electronic Data Capture system in rural plains of Nepal sheds light on the technological and social challenges of mHealth. The study enlisted community women with limited education as nutrition mobilisers to run women's groups providing them with pictorial apps on ‘low-spec' smart phones to record information about women's groups and home visits. More educated data collectors also used smart phones to collect detailed data about women and their babies during pregnancy and after birth. In a few cases nutrition mobilisers' spouses misused the phones, replacing the operating system and wiping memory cards for personal use. Challenges such as scarcity of electricity for charging and limited phone signal also somewhat affected implementation. However, despite the challenges, the intervention made large-scale trial data collection faster and less error-prone.

SafeSIM intervention is the WHO's RMNCH full continuum of care intervention. It uses mobile phones for registration during pregnancy until postnatal counseling visits and is aimed at reducing maternal mortality. It is used to register births and sends FCHVs SMS reminders about when to visit patients for counseling and health education, and expected date of delivery. FCHVs perceived the app to be helpful in simplifying their job. It resulted in an increase in timely ANC visits, treatments and increased awareness on the importance of ANC, which is expected to improve the health status of pregnant women. Providing FCHVs with phones to establish personal ownership, reduced misuse by other family members (68) which was a social challenge in Style et al. (67) study.

Most mHealth interventions focus on using mobiles for Data Entry (69) and communication (70). Some data collecting apps have been used to assist in diagnosis of medical conditions like epilepsy (71), assessing the prevalence of blindness, cataract surgical coverage (72) and collecting clinical data (73). MOH in collaboration with WHO also used mobile phones to monitor measles-rubella vaccination campaigns (74).

Almost all interventions use mobile phones to expand the reach of health care services in remote areas across Nepal's hilly terrain, overcoming some of the challenges of limited infrastructure to access some health services. The above studies provided user's positive opinions on mHealth and demonstrated its potential for improving data quality and security. Problems such as electrical outages and unreliable networks do not signify drawbacks of the interventions but rather the infrastructural deficits in Nepal, which are rapidly being overcome as the demand for mobile phone usage increases.

### mHealth Opportunities in Nepal

In light of the increasing ownership of smartphones together with increasing mobile subscriptions and network coverage across Nepal (30, 33, 34), mHealth may emerge as an effective tool in Nepal's healthcare development. The annual increase in mobile subscriptions and network coverage of more than two-thirds of the nation suggests great potential for new digital interventions in the future. However, some of the increase in subscriptions may be due to individuals having multiple SIM registrations or subscriptions which are non-active today.

Previous studies describe conflicting responses on the accessibility of mobile networks (67, 73, 74), reflecting variability depending on remoteness and network coverage. In spite of this concern, apps like MANTRA can work without an internet connection so only need a connection for the downloading of the app itself. This is similar to Gupta's study where the app data was uploaded when network connection was available (69).

The availability of cheaper brand smartphones, yearly increase in subscriptions and established network coverage may aid mHealth interventions to cover substantial areas of the underserved populations. An increase of mobile projects by numerous organizations in collaboration with Nepal's MOH (62, 68, 74) is an encouraging indicator of mHealth gaining popularity and incorporating such interventions in the national health infrastructure.

Although the literature describes various interventions designed for RMNCH in LMICs, there is sparse research exploring women's experiences and perception of apps designed to tackle maternal or child health in rural Nepal. Additionally, their attitude toward mHealth technology as a means of empowerment and education about health has not been studied yet. We aim to fill these gaps by understanding users' attitudes toward the MANTRA app in rural Nepal and its feasibility, while exploring the extent that participants experienced change in knowledge through the intervention.

## Methodology

### Study Design

MANTRA aimed to increase maternal and child health resilience before, during and after disasters, using mobile technology (75). The MANTRA serious mobile game project was carried out in Nepal after the 2015 earthquakes, with the serious mobile game component led by UCL-IRDR dPHE Centre. The MANTRA project investigated the hazards and risk perceptions with an aim to provide useful information and communication through mobile phones to contribute to maternal and newborn health resilience. The MANTRA team aimed to use culturally appropriate images on a user-friendly interface suitable for all audiences incorporating visuals pertaining to geo-hazards, maternal and child healthcare modules (76). The MANTRA study was approved by University College London Ethics Committee in London, United Kingdom [10547/001], and the Nepal Health Research Council in Kathmandu, Nepal [Reg. No. 105/2017].

Field tests took place in open community spaces, making a single user study difficult. However, this *in-situ* evaluation provided valuable insights into how participants might actually use a serious game (77, 78).

### Study Sites

The data was collected from three locations of the Central Development Region of Nepal and Kathmandu in 2017. Chyamrangbesi and Chandenimandan Village Development Committees (VDCs) in Kavrepalanchok district were the main study areas, while Siddhipur and Imadol in Kathmandu valley, were chosen for field tests of the app, before conducting focused field tests in the two VDCs.

### Study Recruitment and Participants

The field evaluation study comprised 72 participants: 30 FCHVs, 31 women and 11 men. This paper is based on the analysis of nine Focus Group Discussions with the 61 participants that were women and FCHVs. This paper focuses on women's perception of the intervention, although one focus group discussion with men was carried out to gain insight into men's perception of the MANTRA app.

The research participants were recruited using the chain-referral method, or “referrals made among people who share or know of others who possess some characteristics that are of research interest” [(79). p. 141]. Health Research and Social Development Forum (HERD International), the local affiliated NGO, asked contacts in the villages to invite FCHVs and other community members to participate. All participants provided informed written consent.

### Data Collection Method

Each field test comprised a preliminary assessment of participant's understanding of learning objectives, establishing a baseline for knowledge assessment, followed by 10–30 min of gameplay, immediately followed by a post-game knowledge assessment and a focus group discussion (FGD) and interviews. The data collection concluded with a post-assessment of the learning objectives identical to that used at the beginning. These assessments of knowledge change from playing the game are described in Mueller et al. (80).

All FGDs and interviews were facilitated by HERD International researchers, conducted in Nepali using a topic guide, and were audio recorded for transcription and translation into English (16, 80). The topic guide created by the researchers is published in Mueller et al. (16) and includes elements on perceptions, attitudes, user experience, and how the women might use a resource like the MANTRA app (16). Also, details of the co-development process of the game artwork and image pictograms iteratively co-authored with HERD researchers and the local women end users to ensure local appropriateness and understanding is described in detail in Soriano et al. (76).

### Data Analysis Method

Data were analyzed using a qualitative approach to explore participants' subjective experiences (81). Although the main study collected multiple datasets, this paper focuses on nine FGDs with female respondents only as the study focuses on understanding women's perspective on the prototype reproductive and child health app. The FGD method provides the researcher with group interaction dynamics, allowing elaborative and thorough data to be generated (82) but also allows everyone to speak freely “in the safe and familiar context of their own turf” (82).

HERD International researchers were mainly responsible for organizing fieldwork, facilitation, transcription and translation. dPHE researchers were mainly responsible for analysis of data related to the MANTRA serious mobile game app. Reporting was a collaborative effort among the authors.

FGD transcripts were translated into English from Nepali and analyzed using NVivo software (https://www.qsrinternational.com/nvivo-qualitative-data-analysis-software/home/) (83). We used Thematic Analysis to identify themes generated from the organized data (84). This involved “detailed readings of raw data to derive concepts, themes, or a model through interpretations made from the raw data” (85). It provided comprehensive interpretation of the data (86) resulting in a rich understanding of participants' perceptions and their engagement with the app.

**Figure 4:**
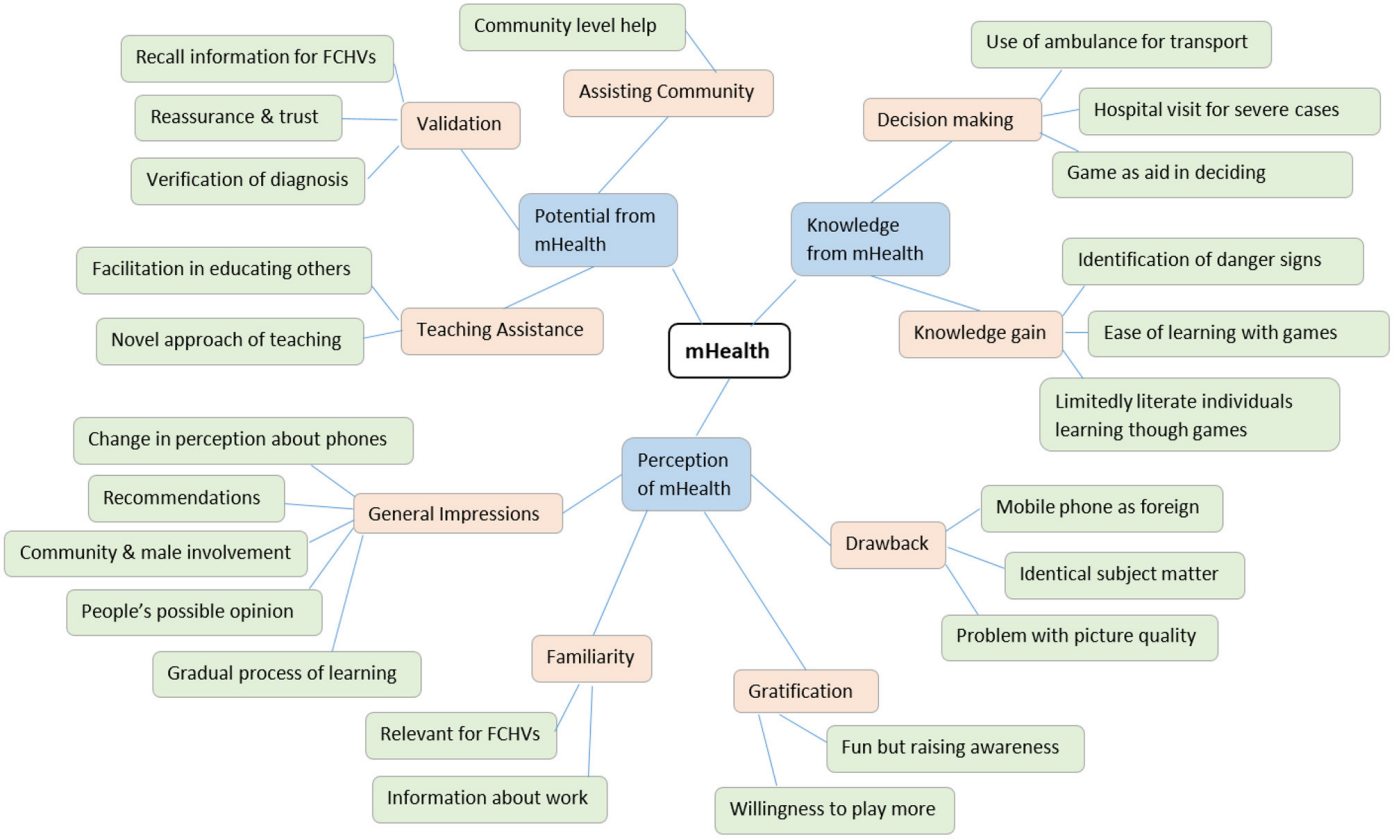
Mind Map to organize data.

We read the transcripts multiple times for familiarization, ensuring the findings reflected participants' perceptions accurately. Transcripts were manually coded into meaningful segments using Nvivo software and basic themes were generated. Emerging relationships and patterns were organized into a mind map (Figure 4) and classified into organizing themes, which led to the emergence of global themes, the principal meanings of the data (87).

## Findings

### Demographics of Participants in Target Audience

Demographic information about participants in the target audience are summarized in Table 1. A total of 61 women participated in nine FGDs comprising 30 FCHVs and 31 community members, ranging from 20 to 60 years of age. Nineteen participants had no education while 42 had some education. Twenty-seven participants owned smartphones, 28 owned basic mobile phones, while 6 did not own phones. Thirty participants were comfortable using smartphones, 24 had previously used apps, and 16 had experienced gaming apps.

**Table 1:** Demographics of participants in target audience.

**Characteristic**	**Group**	***N***	**% of total study participants**
Phone ownership	Smartphone	27	44%
	Basic mobile phone	28	46%
	None	6	10%
Age	18–34 years	21	34%
	35–60 years	40	66%
Education	None or informal	19	31%
	Some education	42	69%
Gender	Female	61	100%
Community role	FCHV	30	49%
	Community Women	31	51%
Village	Chandenimandan	17	28%
	Chyamrangbesi	20	33%
	Imadol	7	11%
	Siddhipur	17	28%

### Thematic Analysis Results

Through the thematic analysis, 24 basic themes emerged from the NVivo codes which were classified into nine organizing themes (see Table 2). The underlying factors and themes led to the identification of the following global themes: Knowledge from mHealth, Potential of mHealth & Perception of mHealth. The column titled *Number of Quotes Found* indicates the collective expression of basic themes.

**Table 2:** Themes derived from the data.

**Global theme**	**Organizing theme**	**Basic theme (NVivo Codes)**	**Number of quotes found**
Knowledge from mHealth	Decision making	Use of ambulance for transport	20
		Hospital visit for severe case	33
		Game as aid in deciding	34
	Knowledge gain	Identification of danger signs	42
		Ease of learning with game	6
		Limited literacy individuals learning through games	15
Potential of mHealth	Assisting community	Community-level help	27
	Teaching assistance	Novel approach of teaching	9
		Facilitation in education others	38
	Validation	Verification of diagnosis	2
		Reassurance and trust	4
		Recall information for FCHVs	7
Perception of mHealth	Gratification	Fun but raises awareness	4
		Willingness to play more	12
	Familiarity	Information from work	7
		Relevant for FCHVs	6
	General impressions	Change in perception about phones	1
		Gradual process of learning	18
		People's possible opinion	29
		Community and male involvement	5
		Recommendations	28
	Hurdles	Mobile phone as foreign	19
		Identical subject matter	2
		Problem with picture quality	10

#### Knowledge From mHealth

The participants went through a process of change in acquiring information and understanding various scenarios, leading to two organizing themes, decision making and knowledge gain.

##### Decision Making

After using the game, the participants were able to identify relevance of certain types of health problem (cases) as “*we were able to learn about which cases we are supposed to give the highest priority. We would be able to identify the health condition of the child in question…We would even consider the cases which need to be taken to the hospital immediately in the ambulance*” (FGD 7-P3). It helped them to prioritize and classify urgency in deciding necessary actions for medical assistance.

##### Knowledge Gain

The app's culturally appropriate images helped in correlating imagery while “*we learnt the different indicators of danger. You could say that we were able to differentiate the dangerous conditions with the help of this game”* (FGD 7-P1). The user-friendly interface and interactive nature aided in understanding scenarios easily “*Actually we only knew about the danger symptoms in pregnant women from the paper picture. We understood more clearly from the game”* (FGD 4-P3). Relating scenarios to corresponding actions signifies an advantage of mobile education in comparison to printed images on posters or paper, as reflected in the statistical analysis of knowledge gain presented by Mueller et al. (80). Finally, an in-depth analysis of women's knowledge retention throughout the game, when the same images are being presented in more complex levels, is presented by Mueller et al. (88).

#### Potential of mHealth

The majority of participants, even the FCHVs, stated the practical possibilities and positive prospects of using the gaming app in their day-to-day life. From this, assisting community, teaching assistance and validation emerged from the global theme.

##### Assisting Community

The participants thought of how they could disseminate the acquired new information within their communities as “*Even though we cannot always be around our friends who know little about these things they will be able to learn about the things that they are supposed to do by looking at the pictures”* (FGD 2-P2). This offers an opportunity for numerous community members to learn essential maternal and child health information and share their learning with others.

##### Teaching Assistance

The FCHV's believe the app “*. would be really good. That would make it very easy for us. We had not seen things in the mobile phone. It was a new and interesting approach*” (FGD 1-P6). Perceiving the phones to be easily accessible compared to paper-based RHMNC materials, the app is considered a novel, innovative and efficient method for teaching as “*The thing is that it increases the speed of the work that we are already doing”* (FGD 4-P1). The mobile app comes in handy especially in rural areas to increase FCHV's efficiency and effectiveness to promote awareness regarding health issues.

##### Validation

FCHV's gained confidence as “*we were able to learn about the different ways to get certain things done. It is not a given that the procedure that we follow to do somethings is always correct. And then, we can learn a new or better alternative from the game. So, that also helped us recall different works that we have done or are doing right now”* (FGD 2-P3). Their ability to verify the diagnosis and re-learn information heightens their zeal and vigor. While “*Some of the local people would not believe the things that we would tell them. So, if they could see the same things in the mobile itself then they would know that we are telling them the right things. So, this would help them trust us more”* (FGD 7-P2). Gaining trust of community members, especially women, is essential for dispersal of knowledge, thus the information offered from a third party source such as the MANTRA app reaffirms FCHVs' efforts while boosting their morale to relate with individuals having pre-notions of certain maternal health issues.

#### Perception of mHealth

Most of the participants viewed the gaming app as a tool for improving RMNCH while a few had diverging opinions. Four organizing themes of gratification, familiarity, general impression and hurdles emerged from the global theme.

##### Gratification

Playing the app game heightens the gamers' interest leading toward better retention of knowledge as “*we got awareness along with the fun of playing the game. We learnt about the things that we are supposed to do”* (FGD 5-P2). Thus, the participants were able to learn complex ideas while enjoying the game, which increased their willingness to play repeatedly and strengthen their knowledge. This was also informally observed at the end of the gaming session by participants stating to each other that they did not want to finish.

##### Familiarity

FCHVs “*.realized that all the things that are there in this game are the works that we have actually been doing”* (FGD 2-P1). They perceived the app's relevance to their existing and past work, and found it reassuring “*… we are female community health volunteers, we find maternal and neonatal health easier to understand”* (FGD 8-P2). Thus, reaffirming their ability and interests to comprehend new technological interventions.

##### General Impression

After playing “*it was easier for us to understand by the medium of pictures. I used to think these games are only meant to be child's play, but today I feel happy to gain this information through game”* (FGD 8-P6). It transformed women's negative views and created curiosity to learn “*we had never seen that mobile game before. But we gradually learnt what we are supposed to do and where we are supposed to go in that game”* (FGD 1-P6). Some believe “*there are some old people who would say that this is something that is unnecessary. The people with old beliefs say such things”* (FGD 2-P3), which makes the older generation hesitant to try new technological interventions. On the other hand, some were optimistic about their elders response: “*. the village is not the same as it was before. They also show interest in learning about these things now”* (FGD 9-P2) suggesting even the older generation may be open to such interventions through awareness and encouragement from their younger family members who have experienced such games.

Such information is vital as “*… Family member are responsible to take care of the patient and they must be aware of all these things. They are the ones to take the patient to the hospital. It is better if the family understand”* (FGD 4-P3). Individuals from the community, especially men, need to acquire such knowledge of danger-signs to take pre-emptive actions. After all, it is generally the husband or family member's responsibility to care for the mother during pregnancy and after birth. “*They would also realize that they have a role to play in the issue of health care in their community. I think that they would develop such feelings.”* (FGD 5-P3) Through dissemination of knowledge from the app amongst a broad group of community members, a collective attitude and communal support system could be built, which would raise awareness within the community and aid in various maternal and child health scenarios.

Positive suggestions for modifications were provided by participants to improve future interventions. For example: “*if we had such audio guidance then it would be definitely easier. The people who are not clear about things will also understand about the things that they are supposed to do in this game. Even the illiterates would be able to play this game”* (FGD 2-P3). Incorporating other relevant health issues as suggested by participants like nutrition, family planning, feminine hygiene etc. in the future would help the app to reach a more of the community. Also, incorporating not only Nepali text but also audio features would help in reaching people across all ages and cultural groups even if they have low literacy.

##### Hurdles

Some participants“*. feel that the pictures need to be clearer for us to understand better.”* (FGD 2-P1). Others expressed that “*It is not that it is difficult. It is simply because we are not used to it.”* (FGD 4-P1). For some, lack of experience with mobile games hindering their clear understanding of the app and cognitive processing of the information. For some women, the mobile was an unfamiliar object since they didn't own- or know how to use- one and they worried about damaging the phone.

## Discussion

Our findings suggest that participants perceived our MANTRA mHealth serious game app intervention as an effective RMNCH tool for healthcare education. Most participants exhibited positive responses to the game. They found it an encouraging opportunity for strengthening the healthcare infrastructure and broadening mHealth reach and awareness in Nepal. A serious game programme has potential of being a constructive device for FCHVs to connect with rural communities through the mode of teaching, community learning and for validation purposes. The MANTRA app provides opportunities for players to acquire information and gain knowledge to make informed decisions in the future. This section discusses the findings in detail, highlighting the significant responses pertaining to the research objective questions.

### Attitudes About MANTRA

Our first objective was to explore the attitudes of women regarding the app. The interactive nature of MANTRA resulted in participants' optimistic response to mHealth. Visual imagery supports ease of comprehension, stimulation of thinking and improves the environment for learning (89). We found similar ease of understanding and constructive engagement with the MANTRA app due to its pictorial nature.

After using the app, women from the communities expressed confidence in processing their health needs. This suggests that the app has potential to help them build independence in handling situations without the assistance of FCHVs and cope without external advice. A reduced need for communication, might raise concerns about misinterpretation of information leading to wrong diagnosis, which could increase the chances of complications and risks.

FCHVs revealed that the MANTRA mobile app aided them to recall their prior knowledge. This is similar to Shorey et al. (78) about utilizing the information in the app for recalling and repeating information. The MANTRA intervention strengthened their confidence in approaching communities and was validating of their existing knowledge and skills. The app had potential to help them develop stronger connections with community members, though the pilot was too short for such benefit to be realized during the study. Similarly, Ilozumba et al. highlighted the importance of strong connections and trust between health workers and communities (54). Recommendations provided by participants included covering additional modules like feminine hygiene, nutrition and family planning, eventually encompassing a larger range of health interventions to correspond with the continuum-of-care approach.

In the male-dominated society of rural Nepal (90), female participants stressed the importance of involving male family members. They believed that it is men's responsibility to look after them during pregnancy and after childbirth.

Although most participants responded positively to the app, a few people from the older generation were less positive, although some respondents over 55 years were open to the new intervention. This correlates with Parker et al. study where some older individuals with limited prior use of mHealth were equally willing to try such methods as younger people (91). This suggests the potential for exploring new approaches to attract older generations to novel interventions. Future research should attempt to understand the perceptions of different generations in order to design and develop an effective mobile intervention.

### Acceptability and Usability

Exploration of participants' acceptability of the MANTRA app and its usability in Nepal was the second objective of this research. Participants identified mHealth as a useful tool in acquiring new knowledge and in encouraging dissemination of the knowledge with others. Prior to mHealth, primary health education and care was predominantly being conducted by FCHVs in women's groups and during health care visits. Tools like the MANTRA app help to increase community awareness and also encourage new members to participate in future interventions. Incorporation of new modules could aid in appealing to male family members to participate and show to concern for their wife and child's health.

Ilozumba et al. (58) use of an effective SMS-based intervention may not be applicable if illiteracy is common, as is the case amongst rural Nepali woman. The interactive approach of MANTRA's visual imagery may have higher chances of significant impact, although participants would have preferred the addition of audio material. Visual imagery aids in the ease of understanding by all, regardless of their educational background. Molnar and Kostkova (41) found that dispersal of information through mobile games led to higher knowledge gain compared to SMS texts. However, a formal usability study was not conducted at the time due to the challenges of women naturally working together rather than individually.

A health intervention dispersed messages of danger signs that led to knowledge increase in community women but not CHWs (54). In the case of MANTRA, both the community women and FCHVs gained knowledge and in addition, FCHVs found the app useful to practice and reaffirm their knowledge. Sharma et al. showed that FCHVs' efforts in improving ANC through the use of mobile phones resulted in an improvement of pregnant women's health (68). Contributing to this broad aim, MANTRA helped community women to develop their personal capacity in identifying risks associated with different scenarios, complementing the FCHVs activities in the community. There is a scarcity of health workers in Nepal (14), which heightens the prospective benefits of mHealth reaching a larger population through mobile devices. However, we also need to consider possible misinformation and misunderstanding by users that may have a negative impact on the women. In a teaching environment, it is possible that some participants might learn the wrong message. This risk could be mitigated in future by FCHVs or other trainers discussing the game and answering participants' questions, further game development to improve clarity, and implementing the game as part of group sessions facilitated by a health worker.

The MANTRA study did not directly encounter some issues and consequences noted in other studies that implemented digital interventions, most probably because the team lent smartphones to participants for the short duration of the field evaluation sessions. User engagement and retention, often being an issue with games interventions studied by Molnar and Kostkova (51) and Molnar and Kostkova (92), was not observed in this study due to the novelty of the smartphone technology to the women and the controlled study set up.

Digital interventions illuminate barriers and social problems. Several barriers from the literature include expense, risk of theft, privacy concerns, limited mobile networks, limited electricity infrastructure, as well as social problems like restrictions on mobile, possibly resulting in difficulties for end users, their households, and the effective implementation of an intervention (10, 64, 78). Mueller et al. (16) discuss some of these considerations as part of the development and localization process of the MANTRA serious mobile game. Several points are particularly relevant to the FGD analysis in this study.

Social and cultural constraints may impede the effectiveness of mHealth interventions in LMICs where patriarchal societal structures affect women's decision-making (93). One possible barrier is the older generation not accepting mobile phones and games among their younger family members and community. Some people may have pre-established negative notions about mobile phones and games having detrimental effects on the community, though this is unlikely in light of the increasing mobile phone usage in Nepal.

Previous studies have described erratic power surges in Nepal as a primary limitation (64, 73). In contrast, MANTRA's participants did not raise concerns on such matters. Rather, they expressed their fears of breaking the phone or not knowing how to use it. Perhaps out approach of lending of phones for a limited period meant that participants were not concerned about recharging phones or other technical problems. Offering RMNCH apps for free could also help eliminate financial concerns of purchasing apps, however limited network coverage or electricity supplies and lack of phone ownership amongst the poorest people may hamper its accessibility.

The issues and unintended consequences discussed are important to consider for larger scale testing of the MANTRA serious game app and implementing similar digital interventions.

### Perception of Knowledge Change

The last objective was to identify women's perception of knowledge change. Our findings suggest that all participants experienced knowledge gain, stating their decisions would now vary depending upon the situations. This finding is further supported by a statistical analysis of the MANTRA app showing statistically significant knowledge improvement in mean scores of the group of participants (75, 80). Identification of danger signs through the app was easy and participant's affinity toward the game was positive. Similar to LeFevre et al. (60), participants showed more interest in acquiring information about danger signs during pregnancy than postpartum.

Although the app incorporated visuals and provided audio cues for right and wrong answers, some women may still interpret scenarios inaccurately. This could delay their decision to seek care when a health problem arose. FCHVs' role as mediators becomes important to clarify scenarios and highlight the importance of gaining correct knowledge. For this reason, mHealth should not replace primary health care workers.

The material in MANTRA that stressed the importance of ambulances, women's awareness to identify emergency situations, and differentiation between health post and hospitals is similar to Sharma et al. (68) which showed an increase of awareness about the importance of care during pregnancy. RMNCH related educational provisions and awareness of risks may lead to an increase in an individual's knowledge, but not necessarily result in behavior change.

The three basic conditions for behavior change are capability, opportunity and motivation. Incorporated with the COM-B model, the Behavior Change Wheel (BCW) (94) describes education as one of the features of intervention, aiding in the formulation of national and international policies for bringing behavior change in individual or communities. Our findings suggest knowledge gain of danger signs could be a motivating factor for women to seek healthcare, but the participant's capacity to make a decision independently may be constrained by social factors. In rural communities, decision-makers are usually elderly males or the mother-in-law (95). A family may also have the motivation to seek care, but the scarcity of health facilities, geographical barriers and economic constraints may hinder their timely action to seek care.

A key element of gaming apps is “fun” which stimulates one's interest and involvement (96) and may result in enhancement of behavior change. Addressing our last objective we found that mHealth brought knowledge change, which could lead to informed decision making. Whilst we hope that this may result in behavior change amongst the participants in the future, assessment of behavior change was beyond the scope of this study. A future study, building on the MANTRA app, should aim to evaluate this.

### Implication and Future Research

MANTRA is the first mixed methods study evaluating a new and open app targeting RMNCH in Nepal. Our study provides new insight on understanding women's perspectives about mHealth and explores the feasibility of mHealth interventions in a rural setting.

Future research may involve continued development of the MANTRA serious game app, as well as a larger study, such as a randomized controlled trial, allowing findings to represent a larger populace. Such research could incorporate individuals across generations and genders while randomly selecting participants from various regions should be implemented for a longer time period with a larger sample size in a non-controlled environment with additional modules. Additionally, the study must examine the linkage between knowledge gain and behavior change of participants incorporating the BCW model or another model of behavior change.

Interventions like MANTRA create positive perceptions of women toward mHealth, can bring knowledge change on RMNCH issues, and support the potential for positive change at an individual level in improving the health outcome of communities. Ultimately, lessons and insights from the MANTRA serious game app may be transferable to other LMIC settings with similar mHealth interventions and support the use of mHealth interventions in innovative and progressive national health agendas.

## Conclusions

This study aimed to understand women's perspectives of the MANTRA serious game app, including attitudes, acceptability and usability using a qualitative approach. The data provide insights on Nepal's use of mobile technologies, mHealth interventions, and challenges. Expanding network coverage and increasing mobile phone subscriptions across Nepal suggest that mHealth apps have great potential to connect with communities in remote terrains. The study highlights women's positive perceptions of mHealth, contextualizes participant's knowledge gain of maternal and child health and increases awareness on informed decision making during maternal and neonatal health problems. Furthermore, MANTRA's utility as an effective teaching and learning tool for FCHVs and rural women supports the use of mHealth interventions and technology to access and learn health information in LMICs.

## Data Availability Statement

The raw data supporting the conclusions of this article will be made available by the authors, without undue reservation.

## Ethics Statement

The studies involving human participants were reviewed and approved by University College London Ethics Committee and Nepal Health Research Council. The patients/participants provided their written informed consent to participate in this study.

## Author Contributions

RK contributed to the design, analysis, interpretation, and drafting of the work. SM contributed to design of the work and drafting of the work. IK contributed to the analysis, interpretation, and drafting of the work. AB contributed to design of the serious game. NS contributed to the conception and design of the work, particularly perinatal health messages, as well as acquisition of field data. AA contributed to the acquisition of field data. MF and SB contributed to conception of the work. AA, NS, and SB assisted to contextualize the content of the game. GH contributed to conception and design of the work and particularly geohazard messages. PY contributed to the interpretation and revising critically for content. PK contributed to conception, design, analysis, interpretation, and drafting of the work. All authors contributed to the article and approved the submitted version.

## Funding

This work was supported by the United Kingdom Research Councils Grand Challenges Research Fund [Project 538621 Award: 173142] NERC Reference: NE/P016103/1.

## Conflict of Interest

GH was employed by the company Hearn Geoserve, Ltd. The remaining authors declare that the research was conducted in the absence of any commercial or financial relationships that could be construed as a potential conflict of interest.

## Publisher's Note

All claims expressed in this article are solely those of the authors and do not necessarily represent those of their affiliated organizations, or those of the publisher, the editors and the reviewers. Any product that may be evaluated in this article, or claim that may be made by its manufacturer, is not guaranteed or endorsed by the publisher.
